# Periocular Infection of *Mycobacterium avium* Complex in a Patient with Interferon-γ Autoantibodies: A Case Report

**DOI:** 10.3390/medicina58070846

**Published:** 2022-06-24

**Authors:** Tzu-Hui Lo, Tou-Yuan Tsai, Lih-Shinn Wang, Tzu-Lun Huang, Nancy Chen

**Affiliations:** 1 Division of Plastic and Reconstructive Surgery, Department of Surgery, Hualien Tzu Chi Hospital, Buddhist Tzu Chi Medical Foundation, Hualien 970, Taiwan; margaretlo1011@gmail.com; 2 School of Medicine, Tzu Chi University, Hualien 970, Taiwan; 96311123@gms.tcu.edu.tw; 3 Department of Emergency, Dalin Tzu Chi Hospital, Buddhist Tzu Chi Medical Foundation, Dalin 622, Taiwan; 4 Division of Infectious Disease, Hualien Tzu Chi Hospital, Buddhist Tzu Chi Medical Foundation, Hualien 970, Taiwan; leonardwang55@gmail.com; 5 Department of Ophthalmology, Far Eastern Memorial Hospital, Banqiao Dist., New Taipei City 220, Taiwan; huang.tzulum@gmail.com; 6 Department of Electrical Engineering, Yuan Ze University, Chung-Li, Taoyuan 320, Taiwan; 7 Departments of Ophthalmology, Hualien Tzu Chi Hospital, Buddhist Tzu Chi Medical Foundation, Hualien 970, Taiwan

**Keywords:** interferon-γ autoantibodies, *Mycobacterium avium* complex, periocular infection

## Abstract

The neutralizing anti-interferon-γ autoantibody (nAIGA)-associated immunodeficiency is an emerging entity frequently associated with the nontuberculosis mycobacterium (NTM) infection and other opportunistic infections. We present a female patient with a mysterious periocular *Mycobacterium avium* complex (MAC) infection, accompanied by sequential opportunistic infections including *Salmollelosis* and herpes zoster infection. Her condition stabilized after long-term antimycobacterial treatment. Nevertheless, neutralizing anti-interferon-γ autoantibody was found in her serum, which was compatible with the scenario of adult-onset immunodeficiency.

## 1. Introduction

The ocular infection of nontuberculosis mycobacterium (NTM), which exist without virulence in the environment, is uncommon. When this occurred, the ocular NTM infections manifested as keratitis, endophthalmitis, dacryocystitis, external adnexal infection, orbital cellulitis, and orbital socket infection [1,2]. A survey of 182 isolates from 142 eyes demonstrated that 63% were associated with biomaterial implants such as orbital prosthesis, scleral buckle, silicone stent, and intraocular lens, whereas the remaining were related to trauma, prior surgery, and steroid use [1]. The most frequent isolate was *M. abscessus/chelonae* complex (83.6%), followed by *M. fortuitum* (12.6%). The *Mycobacterium avium* complex (MAC) isolate was rare, accounting for 1.6% of the ocular NTM infections [1]. Interestingly, in patients with concomitant disseminated NTM and neutralizing anti-interferon-γ autoantibodies, the proportion of MAC isolates was high, at 42% [3]. Therefore, the MAC infection might imply an immunocompromised condition. We report a female patient with opportunistic infections and a poor-healing wound in the medial canthi, which later proved a MAC infection. Years later, her condition of neutralizing anti-interferon-γ autoantibody (nAIGA)-associated immunodeficiency was unveiled after her infections were quiescence after treatment.

## 2. Case Presentation

The 57-year-old female patient, a farmer, presented with persisting and poor-healing ulceration of the right lower eyelid adjacent to the medial canthus for one year.

During the past three years, she suffered from numerous other infections. First, she had Tonsillar Kaposi sarcoma with human herpes virus-8 (HHV-8) infection three years ago, followed by disseminated tuberculosis of the bone in the same year. A year and a half later, she had a *Legionella* pneumophila infection. Eight months ago, she was diagnosed with a mycobacterium tuberculosis infection over the right middle lobe of the lung. In addition, she also suffered from *Salmollelosis* with bacteremia and intermittent herpes zoster infection over S2-3 dermatome for several years. No evidence of human immunodeficiency virus (HIV) infection or malignancy was ever found.

**Figure 1:**
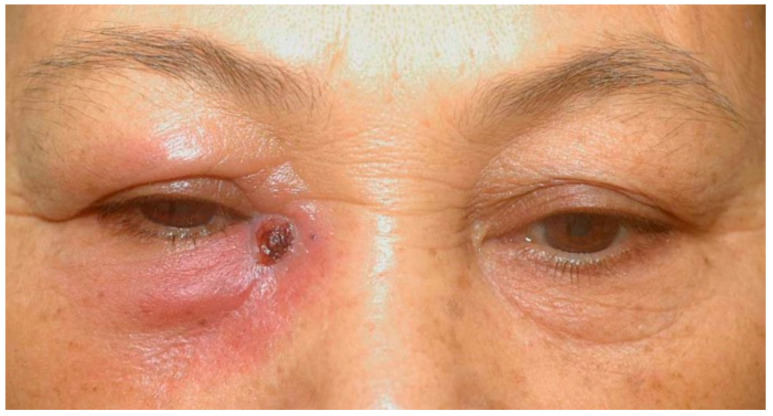
Clinical photograph. The poor-healing wound over the right medial canthal area with periorbital erythematous change and swelling.

**Figure 2:**
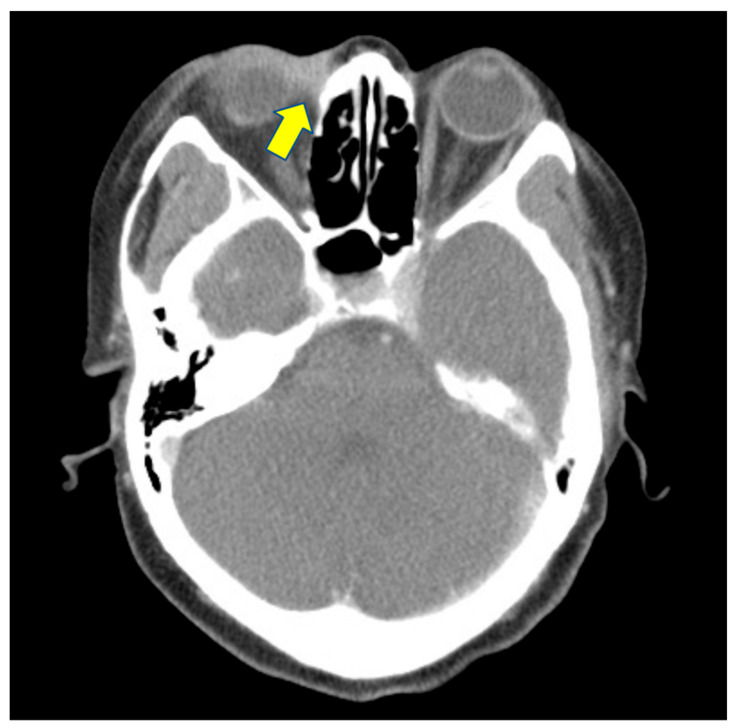
Head and neck computed tomography showed enhanced lesion over the right medial periocular area with involvement of lacrimal sac (arrow).

**Figure 3:**
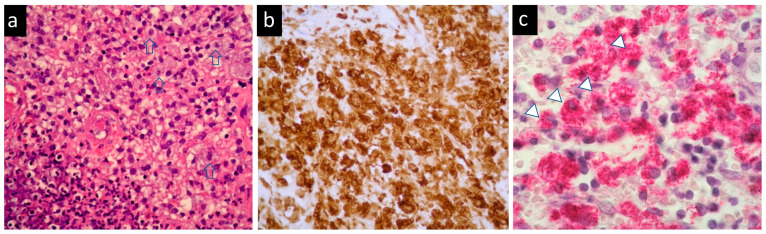
Histopathological studies of the periocular skin biopsy: (**a**) foamy histiocytes (arrow) with granulomatous inflammation (hematoxylin–eosin, original magnification ×100); (**b**) the foamy histiocytes were immunoreactive with CD68, indicating mycobacterium infection (original magnification ×200); (**c**) acid-fast stain showed many *mycobacteria* bacilli (arrowhead) inside the histiocytes (original magnification ×200).

She came to our ophthalmic clinic due to a poor-healing wound overlying the lacrimal sac (Figure 1), accompanied by epiphora and periorbital redness. We irrigated the right nasolacrimal duct and found an obstruction with clear backflow. Other ocular examinations were unremarkable. Laboratory data revealed leukocytosis (13,860/uL) without left shift. Head and neck computed tomography showed enhanced lesion over the right medial periocular area with involvement of the lacrimal sac (Figure 2). Periorbital cellulitis was initially suspected and treated with antibiotics vancomycin and ceftriaxone. However, there was no clinical improvement, whereas wound culture had no growth of bacteria. Biopsy was performed at the ulcerated site, and the pathological studies revealed granulomatous inflammation with a considerable amount of foamy histiocytes and a positive CD-68 stain representing macrophage. Many *mycobacteria* bacilli inside the histiocytes were observed via acid-fast staining (Figure 3). Later, tuberculosis culture results showed *Mycobacterium avium* complex (MAC), a slow-growing nontuberculous mycobacterium (NTM). She was then treated with azithromycin, ethambutol, and rifabutin for a year. The ulcerated wound was healed, but the symptom of epiphora persisted.

Given her history of numerous different infections, including HHV-8-related Kaposi sarcoma, disseminated tuberculosis of the bone, *Legionnaire’s* disease, pulmonary tuberculosis, *Salmollelosis*, and recurrent herpes zoster infection with a periocular injection of MAC this time, we considered the differential diagnoses of immunocompromised conditions associated with atypical infections, for example, T-cell immune deficiencies, lymphoid malignancies, acquired immune deficiency syndrome (AIDS), and neutralizing anti-interferon-gamma autoantibody (nAIGA)-associated immunodeficiency. Eventually, after years of research, autoantibodies against interferon-γ were found in her plasma. Five years into remission, she is currently stable without antibiotics or immunomodulatory agents.

## 3. Discussion

Nontuberculosis mycobacterium (NTM) is typically incubated in the environment and regarded as harmless to healthy subjects. The manifestations of NTM infection-associated diseases depend on host immunity and mycobacterium virulence [4]. In immunocompetent individuals, NTM infections are usually localized. In contrast, disseminated NTM infection, as it involves more than one organ, including the bloodstream, bone, lung, and skin, implies the immunocompromised status of the affected patient. These immunocompromised conditions include malignancy, post-transplant, acquired immune deficiency syndrome (AIDS), and genetic defects that impart Mendelian susceptibility to mycobacterial disease (e.g., deficiencies in the interferon-γ receptor 1 and the IL-12 receptor β1) [4]. Since the report on the neutralizing anti-interferon-γ autoantibody (nAIGA)-associated immunodeficiency, more attention has been drawn to this entity that frequently involves NTM and other opportunistic infections [5].

Although relatively rare, all ocular components can be infected by NTM, causing keratitis, conjunctivitis, scleritis, and endophthalmitis. The cutaneous periocular and adnexal involvement results in cellulitis and dacryocystitis. These NTM infections are predisposed by trauma, surgery, foreign body, and immunocompromised conditions [6]. An excisional biopsy is warranted for treatment and diagnosis when it occurs as persistent cutaneous periocular infections, as occurred in our patient.

*Mycobacterium avium* complex (MAC), comprising two closely related and slow-growing species—*M. avium* and *M. intracellulare*—are the leading causative NTM species in HIV-infected patients [7]; meanwhile, there is a high incidence of blood culture positive in disseminated NTM cases [8]. By contrast, the causative NTM in patients with nAIGA-associated immunodeficiency is variable depending on different geographic areas [9] and ethnicities [4]. Among them, *M. abscessus* and MAC are the most cultured species, and the lymph node is the most frequently involved site [4]. Ocular complications caused by MAC among HIV/AIDS patients are rare and manifest as choroidal or retinal granulomas [10]. To date, no reports have been made of ocular involvement of MAC infection in patients with IFN-γ autoantibodies.

Phagocytosis of mycobacteria is enhanced through the complement bound of the mycobacterial surface, with the response being the production of IL-12, which, in turn, upregulates IFN-γ. Subsequently, IFN-γ activates neutrophils and macrophages to kill intracellular mycobacteria [11]. The anti-IFN-γ-neutralizing antibodies have neutralizing effects against IFN-γ and block the IFN-γ–IL-12 pathway, which is crucial in the host immune system against mycobacterial pathogens [4].

In a study by Browne et al., neutralizing anti-IFN-γ autoantibodies (nAIGA) were detected in 81% of disseminated NTM-infected Asian adults and in 96% of the ones with other opportunistic infections but not found in disseminated tuberculosis, pulmonary tuberculosis, or control group [12]. According to a study by Chi et al., 62.2% of nAIGA-associated immunodeficient patients had a history of *salmonellosis*, while 40.0% had previously contracted herpes zoster infection along with the disseminated NTM infection [13]. Our patient had a history of multiple opportunistic infections, and given the pathological picture of *mycobacteria* bacilli inside the histiocytes, combined with the MAC culture results, the differential diagnosis should have included adult-onset immunodeficiency besides AIDS. However, the diagnosis was challenging; the median time from disease onset to the final diagnosis of disseminated NTM associated with nAIGA was 1.6 years (ranging from 0.25 to 19 years) in the analysis of fifty patients by Wu et al. [3].

Treatments of NTM are based on the infectious species, and the most common combination regimen includes rifampin, ethambutol, amikacin, linezolid, and macrolides [14,15,16]. In addition to antimycobacterial regimens, the rationale for combining immunomodulators stems from the correlation between high-titer IFN-γ autoantibody and the likelihood of long-term opportunistic infections. However, there is no standard regimen of immunomodulatory agents yet. Rituximab, a monoclonal antibody against CD-20 on the B-cell surface could reduce IFN-γ autoantibodies, resume the signaling of IFN-γ, and restore immunity toward intracellular pathogens. It has been a promising treatment for refractory nAIGA-associated infections [17,18,19,20], whereas cyclophosphamide has also been used as an adjunct treatment, with substantial effects and faster achievement of remission [9,21]. Surgical procedures such as drainage or resection have been performed to diagnose and ameliorate local infections [4]. A study comparing patients with NTM infections associated with nAIGA in Thailand and USA showed a decrease in serum nAIGA over time without using immunomodulating agents. The reduction in autoantibodies was not related to antibiotics use, but elevated autoantibodies were consistent with infections [9]. Therefore, researchers advocate that the treatment plan should be guided by clinical manifestations, images of lesions, and nIFN-γ autoantibody levels, aiming to develop an optimal treatment regimen and disease management guidelines [20,22].

Our patient presented with a persistent periocular wound of MAC infection and multiple opportunistic infections during the disease course. The diagnosis of the nAIGA-associated immunodeficiency relies on detecting intracellular infections related to the IFN-γ pathway [20]. Although the patient is now in stable condition without immunomodulating therapy and prophylactic antibiotics, high surveillance and long-term follow-up are mandated. The case reminds us of the importance of keeping this nAIGA-associated immunodeficiency as one of the differential diagnoses and that the cooperation of multidisciplinary specialists is crucial for optimal patient care.

## Data Availability

Not applicable.

## References

[1] Girgis D.O., Karp C.L., Miller D. (2012). Ocular infections caused by non-tuberculous mycobacteria: Update on epidemiology and management. Clin. Exp. Ophthalmol..

[2] Kim A.Y., Davis A.S., Moreau A., Drevets D.A., Melendez D.P. (2020). Management of nontuberculous mycobacterial infections of the eye and orbit: A retrospective case series. Am. J. Ophthalmol. Case Rep..

[3] Wu U.I., Wang J.T., Sheng W.H., Sun H.Y., Cheng A., Hsu L.Y., Chang S.C., Chen Y.C. (2020). Incorrect diagnoses in patients with neutralizing anti-interferon-gamma-autoantibodies. Clin. Microbiol. Infect..

[4] Hase I., Morimoto K., Sakagami T., Ishii Y., van Ingen J. (2017). Patient ethnicity and causative species determine the manifestations of anti-interferon-gamma autoantibody-associated nontuberculous mycobacterial disease: A review. Diagn. Microbiol. Infect. Dis..

[5] Hoflich C., Sabat R., Rosseau S., Temmesfeld B., Slevogt H., Docke W.D., Grutz G., Meisel C., Halle E., Gobel U.B. (2004). Naturally occurring anti-IFN-gamma autoantibody and severe infections with Mycobacterium cheloneae and Burkholderia cocovenenans. Blood.

[6] Moorthy R.S., Valluri S., Rao N.A. (2012). Nontuberculous mycobacterial ocular and adnexal infections. Surv. Ophthalmol..

[7] Varley C.D., Ku J.H., Henkle E., Schafer S.D., Winthrop K.L. (2017). Disseminated Nontuberculous Mycobacteria in HIV-Infected Patients, Oregon, USA, 2007–2012. Emerg. Infect. Dis..

[8] Griffith D.E., Aksamit T., Brown-Elliott B.A., Catanzaro A., Daley C., Gordin F., Holland S.M., Horsburgh R., Huitt G., Iademarco M.F. (2007). An official ATS/IDSA statement: Diagnosis, treatment, and prevention of nontuberculous mycobacterial diseases. Am. J. Respir. Crit. Care Med..

[9] Hong G.H., Ortega-Villa A.M., Hunsberger S., Chetchotisakd P., Anunnatsiri S., Mootsikapun P., Rosen L.B., Zerbe C.S., Holland S.M. (2020). Natural History and Evolution of Anti-Interferon-gamma Autoantibody-Associated Immunodeficiency Syndrome in Thailand and the United States. Clin. Infect. Dis..

[10] Pepose J.S., Holland G.N., Nestor M.S., Cochran A.J., Foos R.Y. (1985). Acquired immune deficiency syndrome. Pathogenic mechanisms of ocular disease. Ophthalmology.

[11] Fulton S.A., Johnsen J.M., Wolf S.F., Sieburth D.S., Boom W.H. (1996). Interleukin-12 production by human monocytes infected with Mycobacterium tuberculosis: Role of phagocytosis. Infect. Immun..

[12] Browne S.K., Burbelo P.D., Chetchotisakd P., Suputtamongkol Y., Kiertiburanakul S., Shaw P.A., Kirk J.L., Jutivorakool K., Zaman R., Ding L. (2012). Adult-onset immunodeficiency in Thailand and Taiwan. N. Engl. J. Med..

[13] Chi C.Y., Lin C.H., Ho M.W., Ding J.Y., Huang W.C., Shih H.P., Yeh C.F., Fung C.P., Sun H.Y., Huang C.T. (2016). Clinical manifestations, course, and outcome of patients with neutralizing anti-interferon-gamma autoantibodies and disseminated nontuberculous mycobacterial infections. Medicine.

[14] Yeh Y.K., Ding J.Y., Ku C.L., Chen W.C. (2019). Disseminated Mycobacterium avium complex infection mimicking malignancy in a patient with anti-IFN-gamma autoantibodies: A case report. BMC Infect. Dis..

[15] Ikeda H., Nakamura K., Ikenori M., Saito T., Nagamine K., Inoue M., Sakagami T., Suzuki H., Usui M., Kanemitsu K. (2016). Severe Disseminated Mycobacterium avium Infection in a Patient with a Positive Serum Autoantibody to Interferon-γ. Intern. Med..

[16] Keragala B., Gunasekera C.N., Yesudian P.D., Guruge C., Dissanayaka B.S., Liyanagama D.P., Jinadasa G.I.M., Constantine S.R., Herath H. (2020). Disseminated Mycobacterium simiae infection in a patient with adult-onset immunodeficiency due to anti-interferon-gamma antibodies—A case report. BMC Infect. Dis..

[17] Czaja C.A., Merkel P.A., Chan E.D., Lenz L.L., Wolf M.L., Alam R., Frankel S.K., Fischer A., Gogate S., Perez-Velez C.M. (2014). Rituximab as successful adjunct treatment in a patient with disseminated nontuberculous mycobacterial infection due to acquired anti-interferon-γ autoantibody. Clin. Infect. Dis..

[18] Koizumi Y., Sakagami T., Nishiyama N., Hirai J., Hayashi Y., Asai N., Yamagishi Y., Kato H., Hagihara M., Sakanashi D. (2017). Rituximab Restores IFN-γ-STAT1 Function and Ameliorates Disseminated Mycobacterium avium Infection in a Patient with Anti-Interferon-γ Autoantibody. J. Clin. Immunol..

[19] Naik R., Cortes J.A. (2016). Persistent Mycobacterium abscessus infection secondary to interferon-γ autoantibodies. Ann. Allergy Asthma Immunol..

[20] King E.M., Weaver V.K., Kestler M.H. (2021). Treatment Dilemmas in Disseminated Nontuberculous Mycobacterial Infections With Interferon-gamma Autoantibodies. Open Forum Infect. Dis..

[21] Laisuan W., Pisitkun P., Ngamjanyaporn P., Suangtamai T., Rotjanapan P. (2020). Prospective Pilot Study of Cyclophosphamide as an Adjunct Treatment in Patients With Adult-Onset Immunodeficiency Associated With Anti-interferon-γ Autoantibodies. Open Forum Infect. Dis..

[22] Chawansuntati K., Rattanathammethee K., Wipasa J. (2021). Minireview: Insights into anti-interferon-gamma autoantibodies. Exp. Biol. Med..

